# Increased reservoir ages and poorly ventilated deep waters inferred in the glacial Eastern Equatorial Pacific

**DOI:** 10.1038/ncomms8420

**Published:** 2015-07-03

**Authors:** Maria de la Fuente, Luke Skinner, Eva Calvo, Carles Pelejero, Isabel Cacho

**Affiliations:** 1 Departament de Biologia Marina i Oceanografia, Institut de Ciències del Mar, CSIC, Passeig Marítim de la Barceloneta 37–49, Barcelona 08003, Spain; 2 Godwin Laboratory for Palaeoclimate Research, Department of Earth Sciences, University of Cambridge, Downing Street, Cambridge CB2 3EQ, UK; 3 Institució Catalana de Recerca i Estudis Avançats, Barcelona, Spain; 4 Grup de Recerca de Geociències Marines, Departament d'Estratigrafia, Paleontologia i Geociències Marines, Universitat de Barcelona, C/Martí i Franquès, Barcelona 08028, Spain

## Abstract

Consistent evidence for a poorly ventilated deep Pacific Ocean that could have released its radiocarbon-depleted carbon stock to the atmosphere during the last deglaciation has long been sought. Such evidence remains lacking, in part due to a paucity of surface reservoir age reconstructions required for accurate deep-ocean ventilation age estimates. Here we combine new radiocarbon data from the Eastern Equatorial Pacific (EEP) with chronostratigraphic calendar age constraints to estimate shallow sub-surface reservoir age variability, and thus provide estimates of deep-ocean ventilation ages. Both shallow- and deep-water ventilation ages drop across the last deglaciation, consistent with similar reconstructions from the South Pacific and Southern Ocean. The observed regional fingerprint linking the Southern Ocean and the EEP is consistent with a dominant southern source for EEP thermocline waters and suggests relatively invariant ocean interior transport pathways but significantly reduced air–sea gas exchange in the glacial southern high latitudes.

## Abstract (web summary)

Accurate deep Pacific Ocean ventilation ages are hindered by poor surface reservoir age reconstructions. Here, the authors generate new ventilation ages for the deep waters of the Eastern Equatorial Pacific, which indicate reduced air-sea gas exchange during the last glacial.

## Graphical abstract

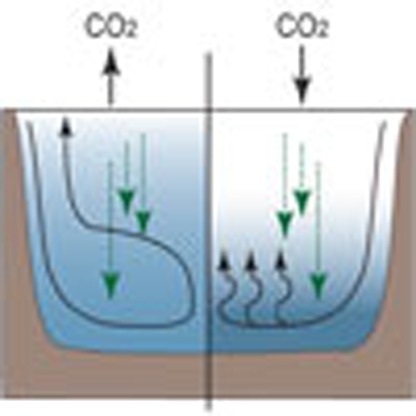

The Pacific accounts for ∼50% of the total ocean volume, such that changes in its average chemistry may exert a significant influence on that of the global average ocean and therefore that of the atmosphere and the Earth's climate. Indeed, it has long been proposed that a reduction in the ocean's average rate of overturn^1^ and/or ocean-atmosphere CO_2_ exchange^2^ may have been the single most important contributor to reduced atmospheric CO_2_ during past glacial periods, and that this would be confirmed by the identification of a significant increase in the average extent of radiocarbon depletion (that is, the ocean-atmosphere radiocarbon disequilibrium or radiocarbon ‘ventilation age') in the Pacific Ocean during the last glacial period^3^. Despite a surge of marine radiocarbon studies focusing on this issue in recent years, and despite circumstantial support for this hypothesis from atmospheric radiocarbon concentration and production reconstructions^4^,^5^,^6^, the glacial radiocarbon inventory of the Pacific Ocean and its evolution across the last deglaciation remain poorly constrained.

Thus, radiocarbon ventilation reconstructions from throughout the mid-depth (∼500–1,500 m) and deep (>1,500 m) Pacific are in apparent conflict, with some indicating very little or no change in radiocarbon ventilation between the Last Glacial Maximum (LGM) and the pre-industrial period^3^,^7^,^8^,^9^,^10^,^11^,^12^,^13^, some indicating transient anomalies of varying magnitude and sense^14^,^15^,^16^,^17^, and with others showing quite significant changes, expressed as a net change between the LGM and the pre-industrial^18^,^19^,^20^,^21^ or as a positive anomaly in the deglaciation^22^,^23^,^24^,^25^ (Table 1). Although much of the apparent conflict between the various Pacific radiocarbon ventilation records may reflect small-scale aspects of the glacial circulation as compared with the present (resulting in highly localized signals or errors in water-mass attribution), it could also reflect to a large extent the different methods and assumptions that are adopted in the various studies.

Perhaps the most important methodological consideration is whether or not independent calendar age control, and therefore surface reservoir age reconstructions are available. It is notable that most of the existing radiocarbon ventilation records from the Pacific are based on the commonly used method of calculating benthic-planktonic radiocarbon age offsets (B–P) (Table 1), which ignores the possible impact of variable surface reservoir ages on the inferred radiocarbon disequilibrium of deep waters relative to the atmosphere (that is, the benthic-atmosphere radiocarbon age offset (B-Atm)). Furthermore, it is also notable that studies adopting this method or the ^14^C projection-age method^26^, and assuming invariant surface reservoir ages, tend not to find evidence of poorly ventilated glacial mid/deep Pacific ocean waters^3^,^7^,^8^,^9^,^10^,^11^,^12^,^13^,^15^,^16^,^17^ (Table 1). However, numerical model experiments have suggested that surface reservoir age should in principle have varied since the last glacial period^27^, and this has been confirmed via a range of techniques applied, not only in the Pacific Ocean^17^,^20^,^21^,^24^, but also in the Southern Atlantic Ocean^28^ and other basins^29^,^30^. Notably, Siani *et al.*^24^ suggested that data from a previous study at a nearby location (core SO161-SL22 (ref. 12)) would indeed show significant changes in ventilation ages over the glacial period if their surface reservoir ages were taken into account. These observations serve to underline the importance of assessing the variability of the surface reservoir ages, especially when using the B–P offset approach to calculate radiocarbon ventilation ages.

Today, the Southern Ocean is the main region where water from the ocean interior makes its first contact with the surface, and where surface water first enters the ocean interior^31^. It is also the main source of deep and abyssal water exported to the rest of the global ocean^32^, and it therefore plays a key role in the water-mass budget and the ventilation (that is, the mixing of surface water properties into the ocean interior) of the Pacific Ocean^33^. It is for this reason that the Southern Ocean has been proposed as the main area for the release of ‘old' carbon-rich deep-water from the ocean interior at the close of the last glacial, possibly due to increased upwelling and/or air–sea gas exchange as a result of altered winds^34^ and/or sea-ice/shallow sub-surface density stratification^28^,^35^. A connection between the high and low latitude Pacific is provided through upwelled shallow sub-surface/intermediate waters from the Southern Ocean (See Methods for further details) in the Eastern Equatorial Pacific (EEP) via the Equatorial Undercurrent (EUC), identified for both the modern^36^,^37^,^38^ and the glacial circulation^34^,^39^,^40^,^41^,^42^. This far-field dynamical connection would imply that changes occurring in the Southern Ocean should exert an influence on the hydrography of the thermocline in low latitude areas like the EEP. The EEP thus provides an excellent study location for assessing past changes in Pacific radiocarbon ventilation, in particular where these are believed to originate in the Southern Ocean.

Here we present deep and shallow sub-surface (that is, deep thermocline) radiocarbon ventilation age reconstructions for the EEP, from sediment core ODP Site 1240 located in the Panama Basin (0°01.31′ N, 86°27.76′ W; 2,921 m water depth). This core site is currently bathed by Upper Circumpolar Deep Water (UCDW) and/or Pacific Deep Water (PDW), with southern sourced shallow sub-surface/intermediate waters contributing to the EEP thermocline via the EUC. Deep-water radiocarbon ventilation ages are estimated from B–P age offsets (B: mixed benthic foraminifera; P: *Neogloboquadrina dutertrei*, a deep thermocline-dwelling planktonic foraminifer, 100–150 m water depth^43^), plus a variable shallow sub-surface reservoir age (yielding B-Atm age offsets), which is derived on the basis of chronostratigraphic alignment to Greenland ice-core records. Notably, the revised chronology that we derive for this core implies a significant increase in both deep-water and shallow sub-surface radiocarbon reservoir/ventilation ages during the last glacial period in the EEP, consistent with a significant change in the radiocarbon concentration of waters exported from the Southern Ocean to the rest of the global ocean during the last glacial period.

## Results

### Radiocarbon dates and B–P offsets in core ODP Site 1240

**Figure 1:**
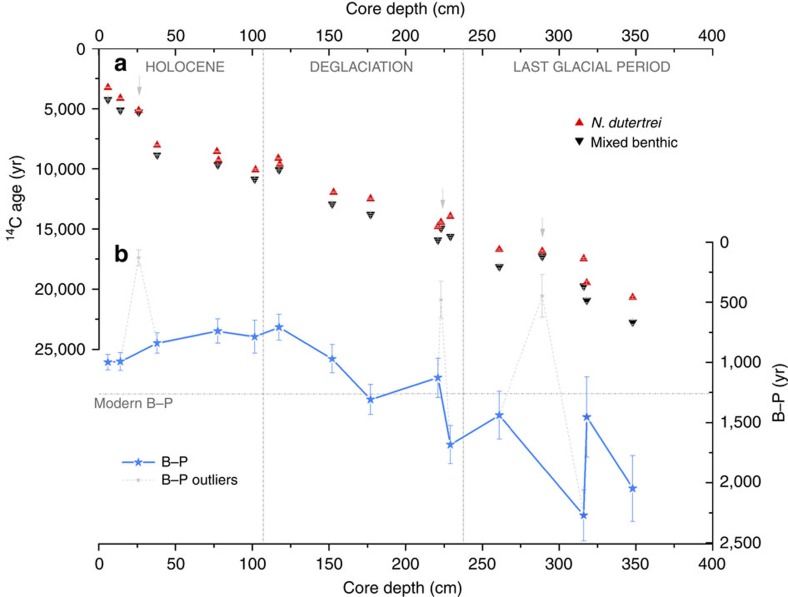
Summary of planktonic and benthic radiocarbon dates and their offsets from core ODP1240. (**a**) Radiocarbon dates from *N. dutertrei* (red triangles) and mixed benthic foraminifera (black triangles) (all dates were δ^13^C-normalized, without reservoir age correction and uncalibrated to calendar years), with associated 1σ-uncertainties. The discarded B–P offsets are indicated by a grey vertical arrow. Note that planktonic dates from intervals 77/78 and 117/118 were averaged for B–P calculation to be able to compare with the coeval benthic radiocarbon dates, which were combined due the low carbonate abundance of each interval (see Methods and Supplementary Table 1). (**b**) Benthic-Planktonic radiocarbon offset (B–P) (thick light blue line and stars) plus the discarded B–P offsets (stapled light grey line) (error bars represent combined 1σ error in ^14^C dates). Modern B–P offset is shown by a dashed horizontal line and relevant climatic periods are separated by dashed vertical lines.

Seventeen paired planktonic and benthic radiocarbon measurements and the offset between them (B–P) are shown in Fig. 1a,b, respectively, and in Supplementary Table 1. Of the data shown in Fig. 1a,b, three B–P pairs (depth intervals 26, 223 and 289; see Fig. 1b in grey) were flagged up as potential outliers in our present study, on the basis that these three pairs yield B–P offsets that fall anomalously far away from the three-point running mean of the data set and that the benthic ages converge on their planktonic counterparts far more rapidly than could be expected due to real changes in deep-ocean circulation/ventilation. However, we show these tentative flyers in Fig. 1a,b, and note that it is possible (if not presently demonstrable) that other studies in the region might in future provide support for their veracity.

The 14 retained radiocarbon B–P pairs (Fig. 1b) indicate a change of ∼870 year between the last part of the last glacial period (average B–P ∼1,805 year) and the Holocene (average B–P ∼935 year, including the modern B–P offset of ∼1,250 year; see Methods for further detail). Across the deglaciation, decreasing B–P offsets indicate enhanced deep-ocean ventilation that peaks in the early Holocene and then drops again to modern values across this period. These results thus provide clear evidence for a drop in deep equatorial Pacific radiocarbon ventilation ages since the LGM.

### Reservoir age assessment and age model

**Figure 2:**
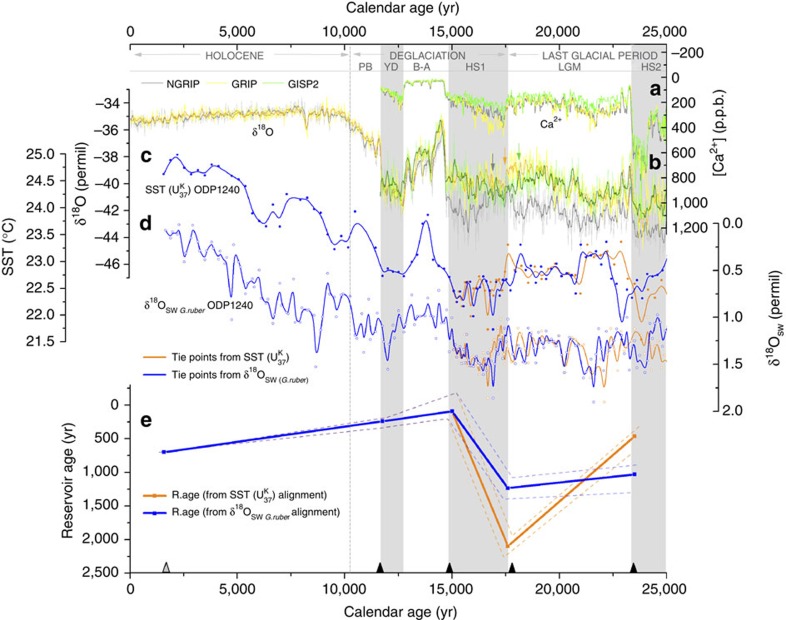
Surface reservoir age assessment for ODP1240 sediment core through the alignment between two independent ODP1240 records and three Greenland ice cores over the last 25 kyr. (**a**) [Ca^2+^] and (**b**) δ^18^O records from Greenland ice cores^44^: NGRIP (grey), GRIP (yellow) and GISP2 (green). Small vertical arrows above Greenland δ^18^O records indicate the apparent HS1 onset for each ice core. (**c**) SSTs from the 
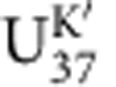
 index and (**d**) δ^18^O_sw (*G. ruber*)_^40^ records from ODP1240 core. In blue, the alignment based on the SST tie-points, and in orange based on the δ^18^O_sw (*G. ruber*)_ tie-points. (**e**) Estimated reservoir age scenarios for ODP1240 core using the two alignments: Thick central lines represent the best reservoir age estimate and stapled lines their upper and lower reservoir age limits (by taking an alignment uncertainty of ±200 year) (in orange, based on the SST correlation, and in blue based on the δ^18^O_sw (*G. ruber*)_ correlation). Selected tie-points are indicated at the bottom by black triangles. An additional core-top date (from the study by Pena *et al.*^40^), corrected and calibrated is also shown (grey triangle). Light grey bands highlight the coldest periods of the last 25 kyr and delimit relevant climatic periods.

An assessment of shallow sub-surface reservoir age variability at the location of ODP1240 (Fig. 2) has been performed by chronostratigraphic alignment between the δ^18^O_ice_ and the [Ca^2+^] records from three Greenland ice cores (NGRIP, GRIP and GISP2 (ref. 44)) placed on the recent GICC05 time scale^45^ and two independent records from Site 1240: (a) Sea Surface Temperature (SST) based on the 
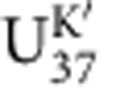
 index (Calvo *et al.*, unpublished) and (b) δ^18^O_sw_ obtained from the planktonic foraminifer *Globigerinoides ruber*^40^. Our proposed alignments are based on a host of previous studies, including previous palaeoclimate reconstructions/interpretations and numerical model studies all of which support the underlying premise of our stratigraphic alignments^46^,^47^,^48^. The outcome of this previous work is that anomalies in sea surface temperatures recorded by alkenones and surface salinities reflected in surface water δ^18^O reconstructions at our location should coincide in time with North Atlantic/Greenland temperature anomalies. There is a set of well tested physical mechanisms underlying this correspondence that relate to changes in cross-equatorial temperature gradients and their impact on easterly winds, equatorial upwelling and moisture transport across the Isthmus of Panama.

For this alignment, four stratigraphic tie-points from the ice-core records were selected coinciding with major climatic changes at the Heinrich Stadial 2 (HS2) end, the HS1 onset, the HS1/Bølling-Allerød transition and the Younger Dryas end (Fig. 2; Supplementary Table 2). The HS1 onset was designated only through the change in the [Ca^2+^] record at 17.6 kyr since precipitation δ^18^O records from the various Greenland ice cores present differences between 19–16 kyr due to different site locations (Fig. 2a,b). An additional age constraint for the ODP1240 core-top was derived based on a single calibrated radiocarbon date (from the study by Pena *et al.*^40^), corrected for the modern shallow sub-surface reservoir age in the area (this is ∼735 year; see Methods for further detail).

On the basis of the above chronologies, two sets of reservoir age estimates were generated (that is, one from each ODP1240 record used for stratigraphic alignment) (Fig. 2e; Supplementary Table 2). These differ due to slight mismatches between the patterns of SST and δ^18^O_sw_ variability before 17 kyr (Fig. 2c,d). Reservoir ages were calculated as the difference between the interpolated ODP1240 planktonic ^14^C for each tie-point^28^ and the atmospheric ^14^C-age (IntCal09 curve^49^) at the calendar age of each tie-point (note that we have not used the more recent IntCal13 (ref. 50) radiocarbon calibration curve to avoid possible problems of inconsistency when comparing with previously published radiocarbon studies that are referenced to IntCal09; only minor differences would arise if IntCal13 were used instead). This approach provides reservoir age estimates only at the four proposed calendar age tie-points. An alternative approach of interpolating calendar ages (and on this basis ^14^C atm) for all the planktonic radiocarbon dates in ODP1240 would yield spurious reservoir ages where no direct calendar age constraints exist (as would occur in the Holocene, for example, where no tie-points were designated).

**Figure 3:**
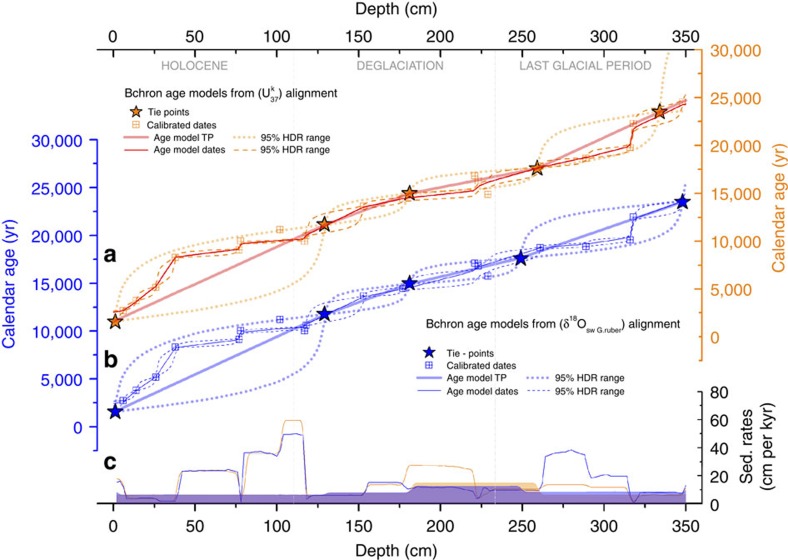
Age models for core ODP1240. Four possible Bchron age models and their uncertainties (95% HDR range) for core ODP1240 derived from; (**a**) two from the δ^18^O_(Greenland ice cores)_—SST_(ODP1240)_ records alignment (orange curves) and (**b**) two from the δ^18^O_(Greenland ice cores)_—δ^18^O_sw (*G. ruber*) (ODP1240)_ records alignment (blue curve). Thick solid lines and stars represent the age models derived only from the tie-points (the error is 200 year). Thin solid lines and squares denote age models using the planktonic radiocarbon measurements corrected for the correspondent interpolated reservoir age obtained from the alignments. (**c**) Sedimentation rates (SR) obtained from the four age models; Shaded areas correspond to SR using only the tie-points and solid lines using the radiocarbon dates. As for **a** and **b**, orange colours refer to the SST alignment, and blue colours refer to the δ^18^O_sw (*G. ruber*)_ alignment.

Four age models were thus derived using the Bayesian age-depth modelling programme Bchron^51^ and the IntCal09 radiocarbon calibration curve^49^ (Fig. 3; Supplementary Table 3): (a) two of them by calibrating 17 *N. dutertrei* radiocarbon dates corrected for the two sets of reservoir age estimates; and (b) two more using only the stratigraphic tie-points (Fig. 3). The purpose of this was to check the consistency of the age models when ignoring the radiocarbon age constraints between stratigraphic tie-points, as well as the radiocarbon age reversals that are automatically rejected by Bchron as outliers in the first two age models. Notably, all of these four chronologies are in good agreement, indicating a good degree of consistency between the various chronostratigraphic constraints that we have adopted, within their respective uncertainty ranges. Only across the Holocene, where no stratigraphic tie-points were selected, do large differences between the radiocarbon and stratigraphic age models emerge, reflecting significant changes in sedimentation rate that cannot be captured by our stratigraphic alignment over this interval. Finally, circumstantial support for the age models is also provided by the fact that deglacial minima in the *N. dutertrei* δ^13^C record from ODP1240 (ref. 40) correspond with similar δ^13^C minima and peak upwelling intensities observed in the Southern Ocean^35^, which also broadly coincide with two prominent minima in the atmospheric δ^13^CO_2_ record (Supplementary Fig. 1).

The two sets of reservoir age estimates show a broadly similar pattern of variability across the last deglaciation, indicating large changes in the shallow sub-surface ocean ventilation over this time interval (Fig. 2e). Quantitatively, small differences are found between both SST and δ^18^O_sw_ alignments, yielding glacial shallow sub-surface reservoir ages of ∼970 years and ∼1,105 years, respectively, that is ∼405 and ∼540 years older than during the Holocene (when reservoir ages are ∼565 years on average, including the modern value of ∼735 years). As shown in Fig. 2e, shallow sub-surface reservoir ages begin to decline coincident with the beginning of the deglaciation (HS1 onset), continuing to drop across HS1 until minimum reservoir ages (that is, maximum ventilation) are reached around the HS1/B-A transition.

### Ventilation ages in the EEP

**Figure 4:**
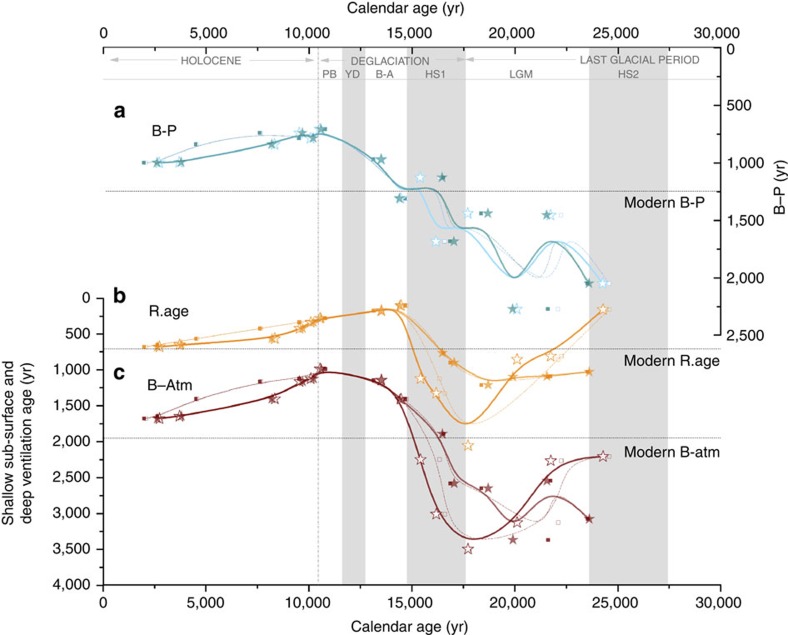
Ventilation scenarios for core ODP1240 over the past 25 kyr using the four estimated age models from Fig. 3 in each case. Age models are b-spline smoothed. (**a**) B–P offset; (**b**) Shallow sub-surface reservoir age; (**c**) Ventilation age (B-Atm offset). Thick solid lines with stars denote age models using the planktonic radiocarbon measurements corrected for the correspondent interpolated reservoir age obtained from the alignments, and thin stapled lines with squares represent the age model derived only from the tie-points (hollow/solid stars and squares corresponds to 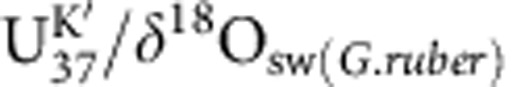 alignments, respectively, in all cases). Modern offsets are shown by dashed horizontal lines. Light grey bands highlight the coldest periods of the last 25 kyr and delimit relevant climatic periods. Note the different *y*-axis scale in **a**–**c**.

Using the observed radiocarbon offsets between B–P (Figs 1b and 4a) and between planktonic-atmosphere (that is, reservoir ages; Figs 2e and 4b), the offset between B-Atm (that is, deep-ocean ventilation; calculated as B-Atm=B–P+R.age; Fig. 4c) can be estimated. Thus, Fig. 4 displays the ventilation history at the location of ODP1240, taking into account all four possible age models and their associated reservoir age implications.

As observed in the shallow sub-surface reservoir age reconstructions, the deep-ocean ventilation (Fig. 4c) also exhibits different amplitude and slightly different trends since the LGM depending on the various alignments (SST or δ^18^O_sw_), but still broadly similar patterns of variability. Quantitatively, the averages from both SST and δ^18^O_sw_ alignments indicate B-Atm values of ∼2,775 years and ∼2,910 years, respectively, during the last part of the glacial period, which would correspond to a ∼1,275 and ∼1,410 years older deep glacial ocean than during the Holocene (on average ∼1,500 years including the modern B-Atm value of ∼1,985 year at 3,000 m water depth; see Methods for further detail). Enhanced ventilation of the deep eastern Pacific is thus observed from the start of HS1, with peak ventilation (minimum B-Atm) being reached at the end of the deglaciation, in the early Holocene (Fig. 4c).

## Discussion

The ventilation reconstructions from Site ODP1240 indicate a glacial-interglacial B–P change of ∼870 years that, taking into account the shallow sub-surface (deep thermocline) reservoir age change of ∼405/540 years (from both SST and δ^18^O_sw_ alignments, respectively), imply a glacial-interglacial B-Atm change of ∼1,275/1,410 years (Fig. 4; Supplementary Table 3). Taken together, these results indicate that both deep and shallow sub-surface reservoir ages (Fig. 4c,b, respectively) were significantly increased at the LGM and that both evolved in a similar manner over the last 25 kyr, *albeit* with a larger change in the deep ventilation ages (as indicated by the change in B–P across the deglaciation; Fig. 4a). Therefore, although the B–P data themselves indicate for the first time a clear deglacial change in deep equatorial Pacific ventilation (Figs 1b and 4a), the B-Atm estimates further suggest that the B–P offsets in fact underestimate this change in deep-ocean ventilation (Fig. 4a,c).

**Figure 5:**
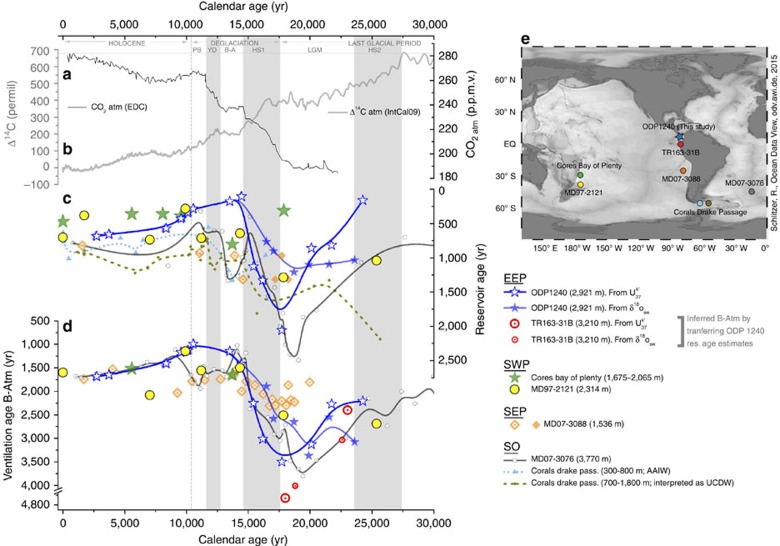
Comparison of reservoir and ventilation age reconstructions from the South Pacific and Southern Ocean over the last deglaciation grouped according to their likely water-mass affiliations. (**a**) Atmospheric CO_2_ concentrations from European Project for Ice Coring in Antarctica (EPICA) Dome C (EDC) ice core (for the deglacial period see ref. 52; for the Holocene period see ref. 68) placed on the age scale of ref. 69. (**b**) Atmospheric radiocarbon activity (Δ^14^C) changes (IntCal09 calibration curve^49^). (**c**) Surface/shallow sub-surface reservoir age records and (**d**) deep-water ventilation reconstructions from several locations in the Pacific and Southern Oceans over the last 25 kyr (B-spline smoothed): ODP1240 (2,921 m; *N. dutertrei*; dark blue hollow/solid stars from SST/δ^18^O_sw_ alignments, respectively; this study); TR163-31B (3,210 m; *N. dutertrei*; red hollow big/small circles from SST/δ^18^O_sw_ alignments in ODP1240, respectively^18^); Bay of Plenty (1,675–2,065 m; *Globorotalia inflata*; green stars^19^); MD97-2121 (2,314 m; *G. inflata*; yellow dots^21^); MD07-3088 (1,536 m; *Globigerina bulloides*; light orange diamonds from^54^,^55^; dark orange diamonds from^24^); MD07-3076 (3,770 m; *G. bulloides* and *G. inflata*; dark grey dots^28^); Corals from the Drake Passage (300–800 m ‘shallow corals'; small light blue triangles and 700–1,800 m ‘deep corals' small dark green dots^57^). Note that the age models used for ODP1240 are those obtained using the calibrated radiocarbon dates and not the tie-points. Note the *y*-axis breaks in **d**. (**e**) Locations of marine records shown in **c**,**d**.

A poorly ventilated glacial shallow sub-surface and deep EEP is thus demonstrated, both by the B–P offsets and B-Atm offsets, with implications for the glacial ocean's total radiocarbon inventory and its role in glacial-interglacial CO_2_ change^6^. Indeed, the radiocarbon ventilation rate of both the thermocline and the deep EEP are observed to increase broadly in time with the onset of deglaciation, the rise in atmospheric CO_2_^52^ and the drop in atmospheric ^14^C^4^,^50^ (Fig. 5a,b). These results would thus support the hypothesized role of reduced ocean-atmosphere CO_2_ exchange rates in sequestering carbon in the glacial ocean interior, and the release of this carbon to the atmosphere across the last termination. The demonstration of reduced radiocarbon ventilation rates in both the deep and shallow sub-surface Pacific Ocean is crucial in this respect, as this basin accounts for ∼50% of the ocean's total volume. Thus, small ventilation or carbon sequestration effects in the Pacific can have a particularly large impact on global ocean chemistry, as well as that of the atmosphere.

The changes in B–P offsets between the LGM and modern observed in ODP1240 clearly reflect a discernable trend in deep equatorial Pacific ventilation across the last deglaciation that is consistent with other B–P records from the northern and southern high latitude Pacific Ocean (southwest Pacific (SWP)^19^,^21^; southeast Pacific (SEP)^24^; northeast Pacific (NEP)^22^). All of these studies indicate slightly higher B–P age offsets during the last glacial period (∼200–600 years) and, although they are not extremely large, they should not be downplayed. Indeed, it has been argued that the full 190‰ drop in atmospheric Δ^14^C since the LGM (ignoring changes in radiocarbon production) could in principle be explained by rather small changes in the mean ventilation age of the whole upper ocean (for example, ∼100 years over 60% of the ocean) if these were combined with a slightly larger change in the mean ventilation of the deepest ocean (for example, ∼3,000 years on average, over 35% of the ocean)^28^.

Although B–P offsets are clearly observed to decrease across the last deglaciation (Figs 1b and 4a), a larger drop in B-Atm offsets is inferred on the basis of our shallow sub-surface reservoir age reconstructions. In a first instance, we explore the regional coherence of our shallow sub-surface reservoir age reconstructions via a comparison of planktonic stable isotope records from our core and core TR163-31B (ref. 18; located in the same area and also with *N. dutertrei* radiocarbon dates), performed by placing each core on its own conventional ^14^C-age scale (Supplementary Fig. 2). For this purpose we use the radiocarbon data from ref. 18. The fact that the isotope stratigraphies and radiocarbon data from the two cores are consistent (Supplementary Fig. 2) indicates that both locations have experienced broadly similar reservoir age changes. On this basis, a ‘regional radiocarbon calibration' can be used to transfer calendar ages (and therefore reservoir ages) from ODP1240 to TR163-31B. This in turn allows B-Atm offsets to be derived for core TR163-31B, using the few available benthic radiocarbon dates from ref. 18, as described below.

While the comparison described above demonstrates that similar reservoir age changes were experienced at the locations of ODP1240 and TR163-31B, it does not provide an independent confirmation of the reservoir ages we derive for ODP1240. However, direct support for our observation of significantly enhanced glacial shallow sub-surface reservoir ages is provided by the few studies in the South Pacific Ocean that have also sought to constrain reservoir age variability using paleoceanographic observations (Fig. 5c). Thus, in the SWP a pioneering study in the Bay of Plenty, using ^14^C-dated tephra, found a considerable increase in shallow sub-surface reservoir ages during the last glacial period^19^ that, with the recently updated age for the Kawakawa tephra^53^, increases the inferred glacial reservoir ages to ∼3,200 years. The latter is an extreme value for a shallow sub-surface radiocarbon reservoir age in the modern context and, although it may yet be proven correct, we tentatively report the Bay of Plenty data in Fig. 5 excluding the glacial point. Indeed, a recent study in the same area (SWP) in core MD97-2121, also using the revised age of the Kawakawa tephra and radiocarbon dates on the same deep dwelling planktonic species (*Globigerina inflata*), found a ∼337 years increase in shallow sub-surface reservoir ages, from 700 to ∼1,037 years, during the last glacial period^21^. The latter values compare well with other reservoir age estimates from core MD07-3088 in the SEP, also based on tephrostratigraphy and marine radiocarbon dates in *G. bulloides*, which indicate ∼500 years older reservoir ages over the latest part of the deglaciation, as compared with modern^54^,^55^. These reservoir age estimates have been extended beyond the range of available ^14^C-dated tephra by assuming an inverse relationship between B–P offsets and planktonic versus benthic foraminiferal Δδ^13^C from the same core, suggesting that increased surface reservoir ages also prevailed during the earlier part of the deglaciation^24^.

**Figure 6:**
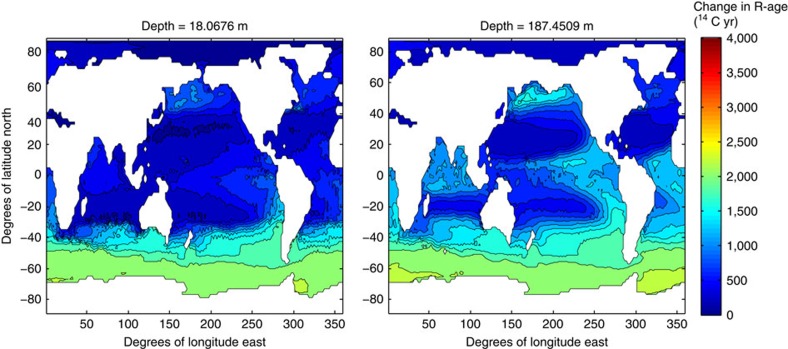
Numerical model simulation. Marine radiocarbon ages (relative to the atmosphere), as reported by Skinner *et al.*^21^, showing ages for 18 and 187 m water depth. The simulation adopts the model framework of DeVries *et al.*^70^ and assumes the modern circulation, *albeit* with >1,000 year surface reservoir ages arbitrarily imposed in the Southern Ocean. The simulation thus demonstrates how an increase in Southern Ocean surface reservoir ages would be transmitted to other regions of the global ocean, including the EEP in particular, simply due to the dominant transport pathways of the ocean circulation. Coherent surface reservoir age changes in the Southern Pacific, Southern Atlantic and EEP could conceivably be explained in this way.

The coherence of these various surface reservoir age estimates^19^,^21^,^24^ (including those from this study) from different regions and water column depths may be explained by the transport pathways that link them together in the modern ocean circulation, as noted by a large number of studies^36^,^38^,^56^. Under the premise that the glacial circulation pathways were not wildly different from the modern (that is, a broadly similar pattern of buoyancy and wind forcing), a numerical model simulation^21^ can be used to show a direct dynamical connection between all these areas and depths (Fig. 6). This illustrates that an increase in the shallow sub-surface reservoir age across the Southern Ocean (for example, as proposed by Skinner *et al.*^28^) should also be conveyed to the shallow sub-surface of the EEP and SWP via the dominant transport pathways in the ocean interior (Fig. 6), as is also indicated for the surface SEP, where *G. bulloides* (dated by Siani *et al.*^24^) currently thrives.

In agreement with this proposition, ODP1240 shallow sub-surface reservoir ages also generally exhibit trends and values that are consistent with reconstructions from the Southern Ocean, including the records based on chronostratigraphically dated planktonic foraminifera from core MD07-3076 from the sub-Antarctic Atlantic^28^ and Drake Passage corals from water depths of 300–800 m and 700–1,800 m (hereafter referred to as ‘shallow corals' and ‘deep corals', respectively)^57^ (Fig. 5c). The planktonic foraminifera from core MD07-3076 would have lived north of the Sub-Antarctic Front where some intermediate waters are subducted and the ‘shallow corals' from the Drake Passage have been interpreted to provide a record of Antarctic Intermediate Water (AAIW) ventilation ages. Interestingly, the ‘deep corals', although initially proposed to reflect the radiocarbon ventilation age of UCDW, are rather similar to the rest of the shallow sub-surface/intermediate water candidate reconstructions (Fig. 5c). This could suggest that, at least over the glacial/deglacial period, the deep Drake Passage corals might have been substantially influenced by SO-intermediate waters, instead of UCDW as previously proposed^57^. Such an interpretation would also help to reconcile the large differences between glacial ventilation ages observed in the deep Drake Passage corals^57^ versus the deep sub-Antarctic Atlantic^28^, which would reflect a large radiocarbon ventilation age gradient between AAIW and LCDW, rather than between UCDW and LCDW.

This consistency between shallow sub-surface reservoir ages in the EEP and the Atlantic and Pacific sectors of the Southern Ocean would support the idea of the proposed link between these two regions through shallow sub-surface/intermediate waters transported by the EUC to the base of the EEP thermocline; an idea that has been advanced previously based on other records^34^,^39^,^40^,^41^. We therefore propose here that the variability we see in *N. dutertrei* from ODP1240 is primarily related to the changing Southern Ocean end-member signature that is being ‘upwelled' in (or at least transported to) the base of the EEP thermocline (where *N. dutertrei* lives), rather than, shifts in *N. dutertrei's* habitat towards the Southern Ocean end-member, for example due to ‘more upwelling'. Nevertheless, we underline that this interpretation is not inconsistent with evidence for variable upwelling in the EEP, or indeed for changes in the contribution of southern sourced water in the EUC^42^. Such changes would only exacerbate the impacts that we infer by making *N. dutertrei* experience sub-surface waters that are more similar to an ‘older/younger' Southern Ocean origin, even if the latter effect was the largest, as we propose on the basis of the regional comparisons and associations shown in Fig. 5 and the numerical model ‘thought experiment' illustrated in Fig. 6.

Regardless of their dynamical associations, or indeed their causes, increased glacial surface/shallow sub-surface reservoir ages have been reconstructed across the South Pacific and Southern Ocean using a range of different approaches, including U-series dating of corals^57^, tephrochronology^19^,^21^,^24^ and chronostratigraphic alignments^28^ as well as radiocarbon ‘plateau tuning' records in the North Pacific^20^. Along with the results of this study, these observations challenge the common assumption of invariant reservoir ages, in particular at high latitudes but also in tropical upwelling areas, as we emphasize here.

In addition to this general agreement of surface/shallow sub-surface reservoir age reconstructions, a broad similarity between deep ventilation age variability across the Equatorial and South Pacific as well as across the Southern Ocean is also apparent (Fig. 5d). Thus, B-Atm estimates from core ODP1240 indicate a significant glacial/interglacial change in radiocarbon ventilation, as also observed in the nearby core TR163-31B when the ‘regional radiocarbon calibration' implied by our chronology for ODP1240 is applied to the few available benthic radiocarbon dates from this core^18^ (Fig. 5d). Similarly, deep-water ventilation changes observed in the SWP^19^,^21^ and the SEP^24^ all show a broadly similar deglacial trend compared with the EEP, as well as comparable B-Atm values. This coherence is highly significant, as it would confirm that a large volume of the ocean interior was indeed very poorly ventilated during the last glacial period; a proposition that has so far been supported by a paucity of data.

The modern deep South and Equatorial Pacific is currently influenced by UCDW and/or PDW, which occupy similar density and depth ranges and are characterized by low oxygen, high nutrients and high ^14^C-age^33^. A hydrographic/dynamical connection between the various ventilation age reconstructions compared in Fig. 5d is therefore entirely plausible; however, it could be seen to conflict with the previous attribution of an AAIW association for core MD07-3088 from the SEP^24^. Alternatively, the radiocarbon ventilation record from 1,536 m in the SEP^24^ may in fact reflect the influence of deeper and older water masses such as PDW/UCDW, instead of AAIW as previously proposed^24^. This speculation is supported by the fact that the SEP core MD07-3088 is currently located just at the boundary between AAIW and PDW. In any event, the deep-water radiocarbon ventilation ages from ODP1240 (this study), and other cores from the deep Eastern Equatorial and South Pacific are all in good agreement with deep radiocarbon ventilation ages from core MD07-3076 from the sub-Antarctic Atlantic^28^ (Fig. 5d), which is consistent with a Southern Ocean source of deep waters to the deep Pacific.

In this study, we have argued that even relatively small changes in B–P radiocarbon ventilation ages are relevant to the question of deglacial radiocarbon and carbon cycling, especially where these are observed over a large ocean volume like the Pacific. Furthermore, due to the possibility (indeed the likelihood) of significant changes in shallow sub-surface reservoir ages since the last glacial period, relatively small deglacial changes in B–P offsets may not necessarily rule out the occurrence of much larger changes in ocean interior radiocarbon ventilation, even in the low latitudes, as we demonstrate here for the EEP. Indeed, our results underline the crucial importance of constraining surface reservoir ages (or radiocarbon-independent calendar ages). Furthermore, these findings are found to be consistent with other surface- and deep-water ventilation age reconstructions from the southern Pacific and Atlantic. This may reflect glacial circulation patterns that were not very different from modern, *albeit* with altered surface boundary conditions (air–sea exchange efficiency) in the Southern Ocean in particular, which we identify as the main source region for poorly ventilated deep glacial waters in the shallow sub-surface and deep EEP. These results have implications for the ocean's role in glacial-interglacial carbon cycling, demonstrating an increase in the Pacific Ocean's mean carbon residence time, which very likely would have contributed to lower atmospheric CO_2_ during the last glacial period. Furthermore, it would appear that this change in the ventilation state of the glacial Pacific may have stemmed in large part from less effective ocean-atmosphere CO_2_ exchange in the Southern Ocean in particular.

## Methods

### Area of study

Sediment core ODP Site 1240 (Ocean Drilling Programme, Leg 202) was collected from the northern flank of the Carnegie Ridge (0°01.31′ N, 86°27.76′ W; 2,921 m water depth) in the southwestern margin of the Panama Basin^58^. This core location is characterized by high-accumulation-rates (>10–15 cm per kyr^58^,^40^), which minimizes possible biases in benthic versus planktonic radiocarbon dating introduced by bioturbation^59^. The Panama Basin presents two main inflow passages for the deepest waters; one along the Ecuador Trench and another one across the broad central saddle of the Carnegie Ridge (sill depths ∼2,900 m and ∼2,300 m, respectively)^60^,^61^. Thus, sediment core ODP1240 must be currently bathed by UCDW and/or PDW (the latter also called Common Water^62^) as both occupy approximately the same density and depth range in the Pacific^33^. Both UCDW and PDW are characterized by low oxygen (especially the UCDW), high nutrients and large ^14^C-age although the origin is different. PDW is formed internally in the Pacific from bottom waters upwelling and diffusion, while UCDW is originated by the mixture of PDW and Indian Deep Water that upwells just below the surface in the Antarctic Circumpolar Current^33^. The shallow sub-surface circulation over our core location is governed by the EUC between 50 and 300 m water depth flowing eastward along the tropics^36^,^37^,^38^. The EUC is partially fed by intermediate water masses (AAIW and Sub-Antarctic Mode Water) originated from upwelled CDW in the Southern Ocean^56^, that are subsequently subducted by winter convection across the Antarctic Polar Front and Sub-Antarctic Front, respectively, spreading its signature into the Pacific interior. The EUC is also partially fed by Sub-Antarctic Water, which is a central water mass originated in the Subantartic zone that subducts at the Subtropical Front (STF; ∼30° S) with its core flowing at 150–200 m water depth within the Subtropical gyre^63^,^64^.

### Sample treatment and analysis

About 19 radiocarbon dates on *N. dutertrei* (a thermocline-dwelling planktonic foraminifer species, thriving at 100–150 m depth^43^) and 17 samples of mixed benthic foraminifera were performed from core ODP1240 (that is, 17 paired B–P radiocarbon dates). *N. dutertrei* was picked from the >212 μm fraction (4–6 mg carbonate per sample), while mixed benthic foraminifera were from the >150 μm fraction (1–6 mg carbonate per sample; adjacent sediment samples were combined when the amount of carbonate was <1 mg).

Sample treatment was performed at the Godwin Laboratory for Palaeoclimate Research of the University of Cambridge (UK). To avoid sample loss, a minimal cleaning approach was carried out: removal of particles, fibres or other detritus or small carbonate clasts with a fine hair brush followed by repeated rinses in deionized water. Cleaned foraminifera were placed into clean glass vials, rinsed with methanol, dried and evacuated prior to graphitization. The calcite was hydrolysed in 0.5 ml of phosphoric acid and then reduced to graphite using a standard hydrogen and iron catalyst method^65^. Primary and secondary standards were graphitized in parallel with samples. NIST OXII was used as a primary reference standard (for normalization), and IAEA-C7 and -C8 were used as secondary standards (for quality control). When samples were <3 mg carbonate, size-matched aliquots of radiocarbon-dead Iceland Spar calcite were graphitized for small sample background (modern contamination) correction^66^. The radiocarbon analyses were carried out at The ^14^CHRONo Centre of the Queen's University of Belfast (UK) using a National Electrostatic Corporation (NEC) compact model 0.5 MV accelerator mass spectrometer, with online δ^13^C measurement.

### Chronology

The ODP1240 core chronology for the last 25 kyr was rebuilt in this study. The previous age model for this core^40^ was based on calibrated radiocarbon dates corrected, as is customarily done, for a constant surface reservoir age of ∼472 years. However, in this study we assessed the variability of shallow sub-surface reservoir ages using independent chronostratigraphic constraints. We found that shallow sub-surface reservoir ages varied significantly over the last 25 kyr and produced new age models that take account of this variability, using the Bayesian depth-age modelling programme Bchron^51^ and the IntCal09 radiocarbon calibration curve^49^.

### Radiocarbon calculations in modern water column

The modern radiocarbon values for ODP1240 core site were obtained from section P19 of the World Ocean Circulation Experiment^63^, very close to our core site. Using the ‘Δ^14^C_natural_' and applying the equation of Stuiver and Polach^67^ (*t*=−8,033ln(1+(Δ^14^C/1,000)), we calculated the current shallow sub-surface and deep ventilation ages in this site. Thus, the ventilation ages would summarize as: modern shallow sub-surface ventilation age (reservoir age) at ∼150 m water depth is ∼735 radiocarbon years; modern deep ventilation age (B-Atm) at ∼3,000 m water depth is ∼1,985 radiocarbon years; modern B–P radiocarbon offset between 150 and 3,000 m water depth is ∼1,250 radiocarbon years.

## Additional information

**How to cite this article**: de la Fuente, M. *et al.* Increased reservoir ages and poorly ventilated deep waters inferred in the glacial Eastern Equatorial Pacific. *Nat. Commun.* 6:7420 doi: 10.1038/ncomms8420 (2015).

## Supplementary Material

Supplementary Information Supplementary Figures 1-2, Supplementary Tables 1-3 and Supplementary References

## Figures and Tables

**Table 1:** Summary of radiocarbon ventilation records from the Pacific Ocean over the last deglaciation grouped by water depth (>1,500 m/<1,500 m) and area.

**Area**	**Coordinates**		**Core name**	**Water depth (m)**	**Study**	**Method to calculate deep ventilation age**	**Reservoir age variability assessment?**	Type of deep ventilation reconstructions*
*Sediment cores <1,500 m water depth*								
EEP	0.5° S	82.1° W	VM19–27	1,370	8	B–P	No (constant R.age)	0
	1°13′ S	89° 41′ W	MV21–30	617	16	B–P	No (constant R.age)	0+
SEP	36°13′ S	73° 40′ W	SO161-SL22	1,000	12	B–P	No (constant R.age)	0
NEP	23.5° N	111.6° W	MV99-MC19/GC31/PC08	705	15	Proj.age	No	0+
NWP	41°07.1′ N	142° 24.2′ E	MR01-K03 PC4/PC5	1,366	14	Proj.age	No	0+
	52° N–56° N	144° E–169° E	Several cores from Bearing Sea and Okhotsk Sea	630–1,765	25	B–P	No (constant R.age)	1+
	34° N	137° E	Several cores from off the Tokai area	1,076–1,356	23	B–P	Yes (TEPHRAS)	1+
								
*Sediment cores >1,500 m water depth*								
EEP	3°37.2′ S	83° 58′ W	TR163-31B	3,210	18	B–P	No (constant R.age)	1
	1.5° S	85.8° W	RC11–238	2,570	8	B–P	No (constant R.age)	0
	5.5° S	106.8° W	VM21–40	3,180	8	B–P	No (constant R.age)	0
	0° 01.31′ N	86° 27.76′ W	ODP1240	2,921	This study	B–P	Yes (chronostratigraphy)	1+
SEP	46° S	75° W	MD07-3088	1,536	24	B–P	Yes (TEPHRAS, partially)	1+
SWP	40° 2.935′ S	177° 59.68′ E	MD97-2121	2,314	21	B–P	Yes (TEPHRAS)	1
	36° S–45° S	177° E–179° E	Several cores from Bay of plenty and Chatman Rise	1,300–2,700	19	B–P	Yes (TEPHRAS)	1
NEP	54.37° N	148.45° W	ODP 887	3,648	22	B–P	No	1+
	42.1° N	125.8° W	W8709A-13PC	2,710	17	Proj.age	Partially, only after 15 kyr	0+
NWP	18° 54′ N	115° 46′ E	Sonne 50–37KL	2,695	3	B–P	No (constant R.age)	0
	36° 04′ N	141° 49′ E	MD01–2420	2,101	13	Proj.age	No	0
	51° 27′ N	167° 73′ E	MD01–2416	2,317	20	B–P	Yes (^14^C Plateau tuning)	1
	20° 07′ N	117° 23′ E	GIK 17940	1,727	20	B–P	Yes (^14^C Plateau tuning)	1
	7° 12′ N	112° 5′ E	V35–5	1,953	7	B–P	No (constant R.age)	0
WEP	6° N	126° E	MD98–2181	2,100	9	B–P	No (constant R.age)	0
	1° S	146° E	MD98–2138	1,900	9	B–P	No (constant R.age)	0
	1.1° N	130° E	MD01–2386	2,820	10,11	B–P	No (constant R.age)	0
CEP	10° 17′ S	111° 20′ W	TT154–10	3,225	7	B–P	No (constant R.age)	0

B–P, benthic-planktonic radiocarbon offset method; CEP, Central Equatorial Pacific; EEP, Eastern Equatorial Pacific; LGM, Last Glacial Maximum; NEP, North East Pacific; NWP, North West Pacific; Proj.age, projection-age method from ref. 26; SEP, South East Pacific; SWP, South West Pacific; WEP, Western Equatorial Pacific.

^*^0 indicates little or no change in radiocarbon ventilation between the LGM and the pre-industrial period; 0+—transient anomalies of varying magnitude and sense; 1—quite significant changes, expressed as a net change between the LGM and the pre-industrial period; 1+—quite significant changes, expressed as a net change between the LGM and the pre-industrial period but with a positive anomaly in the deglaciation.
