# Supplementary material for: Increased reservoir ages and poorly ventilated deep waters inferred in the glacial Eastern Equatorial Pacific
Source: Nat Commun. 2015 Jul 3;6:7420. doi: 10.1038/ncomms8420 (PMC4507014; doi:10.1038/ncomms8420)
Supplement: Supplementary Information — Supplementary Figures 1-2, Supplementary Tables 1-3 and Supplementary References [file ncomms8420-s1.pdf]

Supplementary Figures

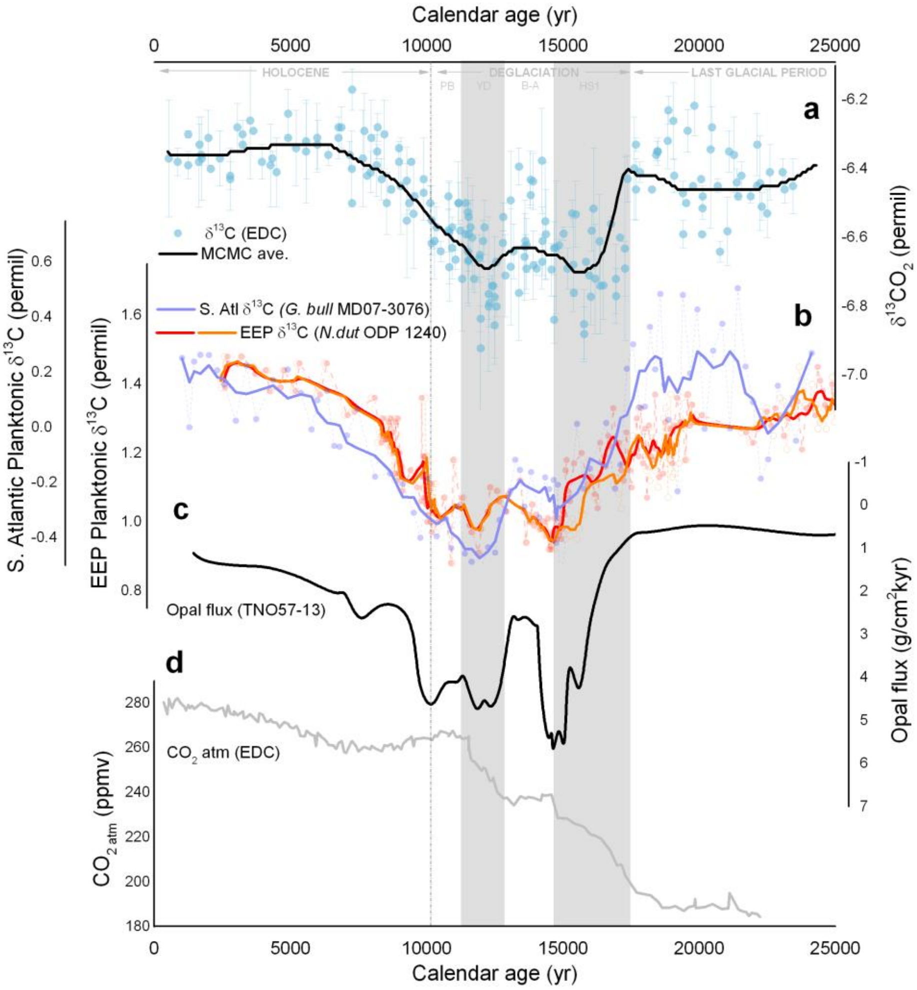

Supplementary Figure 1: Comparison of marine and atmospheric $\delta^{13}C$ records. (a) Atmospheric $\delta^{13}CO_2$ record compilation[1]. (b) *N. dutertrei* $\delta^{13}C$ record from ODP1240[2] placed on the revised age models in this study (red/orange dashed lines with solid/hollow dots; from SST/$\delta^{18}O_{sw}$ alignments, respectively) and *G. bulloides* $\delta^{13}C$ record from MD07-3076[3] from the sub-Antarctic Atlantic (blue dashed line). Solid thick lines indicate the 5-point running averages. (c) TN057-13 opal flux record[4]. (d) Atmospheric CO_2 concentrations from European Project for Ice Coring in Antarctica (EPICA) Dome C (EDC) ice core (for the deglacial period[5]; for the Holocene period[6]) placed on the age scale of[7].

The *N. dutertrei* $\delta^{13}C$ record from ODP1240 (our study) is seen to be highly consistent with the *G. bulloides* $\delta^{13}C$ record[3] from the sub-Antarctic Atlantic and the opal flux record[4], in showing

two periods of minimum $\delta^{13}C$ values that broadly coincide with periods of CO_2 rise and with minima in the atmospheric $\delta^{13}CO_2$ record[1]. This general agreement is what one would expect based on the canonical view that all these records reflect the release of 'isotopically light' carbon from the deep ocean to the atmosphere via the Southern Ocean and the EEP (e.g.[8]). However, it is also apparent from this figure that when considering the records in detail, the marine records are apparently in closer agreement with each other than they are with the atmosphere. In particular, the marine records agree in showing the first occurrence of minimum $\delta^{13}C$ values very late in the Heinrich Stadial 1. Notably, this is apparent in both marine $\delta^{13}C$ records, as well as the opal flux record[4].

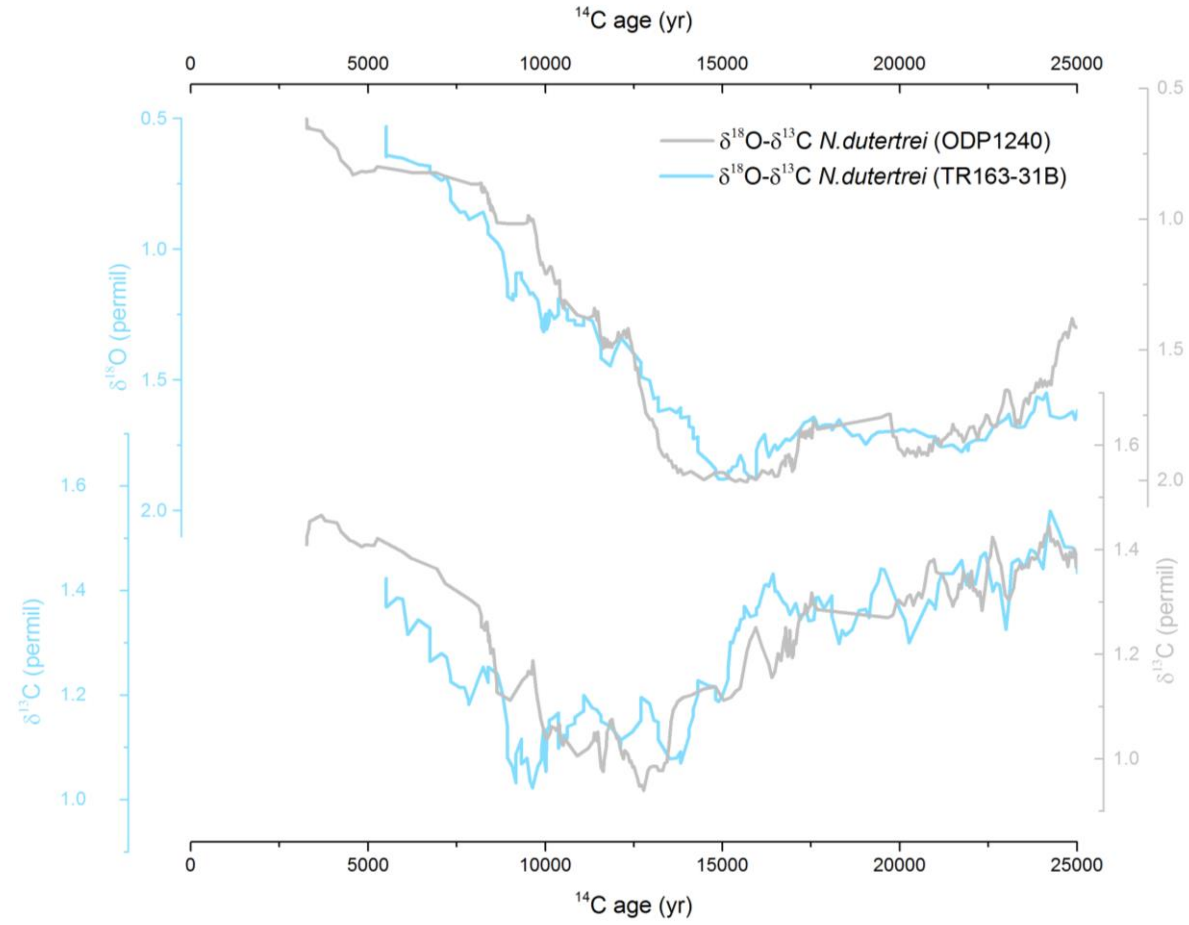

Supplementary Figure 2: Stable isotopes comparison between ODP1240/TR163-31B[9] cores, each one placed on its own radiocarbon age scale. Upper panel displays $\delta^{18}O$ records and lower panel $\delta^{13}C$ records (in grey from ODP1240 and in light blue from TR163-31B[9]). A pretty good match between the stable isotope records from both cores once they are placed on their own conventional ^{14}C age-scales, strongly suggests that both sites have experienced similar shallow sub-surface reservoir age changes. This approach provides a 'regional radiocarbon calibration', and therefore a means to transfer our reservoir age estimates for ODP1240 to TR163-31B (this is done by transferring the calendar ages obtained for ODP1240 ^{14}C dates in the present study).

Supplementary Tables

Benthic dates summary				Planktic dates summary				B-P		
**Depth Cm**	**^{14}C age (benthic) ^{14}C yr**	**±1σ ^{14}C yr**	**Species**	**Depth cm**	**^{14}C age (planktonic) ^{14}C yr**	**±1σ ^{14}C yr**	**Species**	**Depth cm**	**B-P ^{14}C yr**	**±1σ *** ^{14}C yr**
6	4,242	38	mixed benthics	6	3,243	27	*N.dutertrei*	6	999	65
14	5,130	48	mixed benthics	14	4,136	27	*N.dutertrei*	14	994	75
26	5,285	33	mixed benthics	26	5,154	32	*N.dutertrei*	*26*	*131*	*65*
38	8,885	47	mixed benthics	38	8,046	37	*N.dutertrei*	38	839	84
				77	8,583	45	*N.dutertrei*			
				78	9,292	43	*N.dutertrei*			
77.5	9,677	57	mixed benthics	77.5	*8,938	44	*N.dutertrei*	77.5	740	101
**101.5	10,875	76	mixed benthics	**102	10,089	61	*N.dutertrei*	101.5	786	137
				117	9,145	44	*N.dutertrei*			
				118	9,631	46	*N.dutertrei*			
117.5	10,095	62	mixed benthics	117.5	*9,388	45	*N.dutertrei*	117.5	707	107
**152	12,934	64	mixed benthics	**153	11,964	55	*N.dutertrei*	152	970	119
177	13,801	67	mixed benthics	177	12,492	58	*N.dutertrei*	177	1,309	125
221	15,922	86	mixed benthics	221	14,794	77	*N.dutertrei*	221	1,128	163
223	14,933	83	mixed benthics	223	14,454	70	*N.dutertrei*	*223*	*479*	*153*
229	15,630	79	mixed benthics	229	13,947	79	*N.dutertrei*	229	1,683	158
261	18,152	107	mixed benthics	261	16,713	91	*N.dutertrei*	261	1,439	198
289	17,307	91	mixed benthics	289	16,859	86	*N.dutertrei*	*289*	*448*	*177*
316	19,756	118	mixed benthics	316	17,485	94	*N.dutertrei*	316	2,272	212
318	20,946	178	mixed benthics	318	19,492	154	*N.dutertrei*	318	1,454	332
348	22,753	159	mixed benthics	348	20,705	116	*N.dutertrei*	348	2,049	275

Supplementary Table 1. **Compiled radiocarbon data from ODP1240 core.** Benthic (mixed) and planktonic (*N. dutertrei*) radiocarbon dates (δ^{13}C-normalised, without reservoir age correction and uncalibrated to calendar years), and B-P offsets from core ODP1240. *These dates represent the average of two consecutive intervals (77/78 and 117/118) in order to be able to compare with the benthic dates, where two consecutive intervals were combined due the low carbonate abundance of each interval (see Methods). **These intervals are not exactly the same for planktonic and benthic foraminifera, but immediately consecutive. ***The B-P radiocarbon errors represent the addition of planktonic and benthic 1σ radiocarbon errors. Note that the discarded B-P offset intervals are marked in italics and highlighted in grey (See also Results and Fig. 1).

TIE-POINTS SUMMARY				^{14}C PLANKTONIC**	RESERVOIR AGE ESTIMATES								
					BEST ESTIMATE			LOWER ESTIMATE			UPPER ESTIMATE		
Tie-points ODP1240 (depth) cm	Tie-points ICE CORES (cal. age) yr	Event	Source	Interp from ODP 1240 planktonic dates yr	Cal. Age from ICE CORES yr	^{14}C atm Interp from IntCal09 yr	R. Age yr	Cal. Age (-200 yr) yr	^{14}C atm Interp from IntCal09 yr	R. age yr	Cal. Age (+200 yr) yr	^{14}C atm Interp from IntCal09 yr	R. age yr
1	1,560	ODP1240 core-top ^{14}C date *		-	-	-	735***	-	-	735***	-	-	735***
129	11,760	YD end	SST/$\delta^{18}O_{sw}$	10,364	11,760	10,124	240	11,560	10,017	347	11,960	10,177	187
181	15,000	HS1-BA transition	SST/$\delta^{18}O_{sw}$	12,701	15,000	12,609	92	14,800	12,499	202	15,200	12,883	-182
249	17,600	HS1 onset	$\delta^{18}O_{sw}$	15,676	17,600	14,440	1,236	17,400	14,275	1,401	17,800	14,596	1,080
259	"	"	SST	16,540	"	"	2,100	"	"	2,265	"	"	1,944
334	23,500	HS2 end	SST	20,139	23,500	19,675	464	23,300	19,401	738	23,700	19,817	322
348	"	"	$\delta^{18}O_{sw}$	20,705	"	"	1,030	"	"	1,304	"	"	888

Supplementary Table 2. Reservoir age estimates for ODP1240. Selected tie-points from the alignment between $U^{K'}_{37}/\delta^{18}O_{sw\ (G.\ ruber)}$ and $\delta^{18}O_{(Greenland\ ice\text{-}cores)}$ and estimated shallow sub-surface reservoir ages (from the calendar ages obtained from the tie-points (BEST ESTIMATE) and with an error of ±200 yr (UPPER AND LOWER ESTIMATE)). *^{14}C date[2] corrected for modern reservoir age = 700 yr and calibrated, using the IntCal09 calibration curve[10], by Bchron[11]. ** The interpolated planktonic ^{14}C from ODP1240 dates is the same for all reservoir ages estimates (BEST, LOWER AND UPPER) since the core depths do not vary. All planktonic dates were used for the planktonic ^{14}C interpolation since they did not present any apparent anomaly. ***Modern shallow sub-surface reservoir age at 150 m water depth at ODP1240 site (See Methods).

SUMMARY OF ODP1240 DATES						B-P, R.ages and B-Atm ESTIMATES							AGE MODELS			
						B-P		R. ages		B-Atm			From stratigraphic tie-points		From plk^{14}C corr. for the 2 R.age sets	
Depth ODP1240	^{14}C age	±1σ	Depth ODP1240	^{14}C age	±1σ			(SST)	($\delta^{18}O_{sw}$)	(SST)	($\delta^{18}O_{sw}$)		(SST)	($\delta^{18}O_{sw}$)	(SST)	($\delta^{18}O_{sw}$)
	Mixed benthics			Planktonic (*N.dutertrei*)		B-P	±1σ	R.age	R.age	B-Atm	B-Atm	±1σ*	Cal. age	Cal. age	Cal. age	Cal. age
cm	^{14}C yr	^{14}C yr	Cm	^{14}C yr	^{14}C yr	^{14}C yr	^{14}C yr	yr	yr	yr	yr		yr	yr	yr	yr
6	4,242	38	6	3,243	27	999	65	682	682	1,681	1,681	65	2,061	1,976	2,729	2,621
14	5,130	48	14	4,136	27	994	75	653	653	1,647	1,647	75	2,687	2,616	3,705	3,793
38	8,885	47	38	8,046	37	839	84	567	567	1,406	1,406	84	4,563	4,502	8,337	8,201
77.5	9,677	57	77.5	8,938	44	740	101	425	425	1,165	1,165	101	7,651	7,619	9,534	9,688
101.5	10,875	76	102	10,089	61	786	137	339	339	1,125	1,125	137	9,525	9,512	10,085	10,215
117.5	10,095	62	117.5	9,388	45	707	107	282	282	989	989	107	10,776	10,774	10,532	10,590
152	12,934	64	153	11,964	55	970	119	175	175	1,145	1,145	119	13,140	13,118	13,504	13,511
177	13,801	67	177	12,492	58	1,309	125	104	104	1,413	1,413	125	14,728	14,674	14,455	14,395
221	15,922	86	221	14,794	77	1,128	163	1,122	765	2,250	1,893	163	16,325	16,521	15,410	16,465
229	15,630	79	229	13,947	79	1,683	158	1,328	899	3,011	2,582	158	16,594	16,840	16,171	17,042
261	18,152	107	261	16,713	91	1,439	198	2,056	1,211	3,495	2,650	198	17,759	18,355	17,710	18,690
316	19,756	118	316	17,485	94	2,272	212	856	1,096	3,128	3,368	212	22,085	21,598	20,096	19,890
318	20,946	178	318	19,492	154	1,454	332	813	1,092	2,267	2,546	332	22,243	21,715	21,727	21,538
348	22,753	159	348	20,705	116	2,049	275	158	1,030	2,207	3,078	275	24,550	23,520	24,257	23,594

Supplementary Table 3. Summary of the radiocarbon dates selected for the estimations of B-P, reservoir ages (R.age) and deep ventilation ages (B-Atm) from the two alignments and the four estimated age models. Note that the discarded intervals in Supplementary Table S1 are not represented in this supplementary table. However, all planktonic dates were used for the planktonic ^{14}C interpolation and for the age models since they did not present any apparent anomaly. (SST) and ($\delta^{18}O_{sw}$) represent data estimated from the SST and $\delta^{18}O_{sw}$ alignments, respectively. * These radiocarbon errors represent the addition of planktonic and benthic 1σ radiocarbon errors.

Supplementary References

1. Schmitt, J. *et al.* Carbon isotope constraints on the deglacial CO_2 rise from ice cores. *Science* **336,** 711–4 (2012).
2. Pena, L., Cacho, I., Ferretti, P. & Hall, M. A. El Niño–Southern Oscillation–like variability during glacial terminations and interlatitudinal teleconnections. *Paleoceanography* **23,** 1–8 (2008).
3. Skinner, L., Waelbroeck, C., Scrivner, a. E. & Fallon, S. J. Radiocarbon evidence for alternating northern and southern sources of ventilation of the deep Atlantic carbon pool during the last deglaciation. *Proc. Natl. Acad. Sci.* 1–5 (2014).
4. Anderson, R. *et al.* Wind-Driven Upwelling in the Southern Ocean and the Deglacial Rise in Atmospheric CO_2. *Science.* **323,** 1443–1448 (2009).
5. Monnin, E. *et al.* Atmospheric CO_2 concentrations over the last glacial termination. *Science* **291,** 112–4 (2001).
6. Flückiger, J., Monnin, E., Stauffer, B., Schwander, J. & Stocker, T. F. High-resolution Holocene N_2O ice core record and its relationship with CH_4 and CO_2. *Global Biogeochem. Cycles* **16,** (2002).
7. Lemieux-Dudon, B. *et al.* Consistent dating for Antarctic and Greenland ice cores. *Quat. Sci. Rev.* **29,** 8–20 (2010).
8. Spero, H. J. & Lea, D. The cause of carbon isotope minimum events on glacial terminations. *Science* **296,** 522–5 (2002).
9. Shackleton, N. *et al.* Radiocarbon age of last glacial Pacific deep water. *Nature* **335,** 708–711 (1988).
10. Reimer, P. J. *et al.* IntCal09 and marine09 radiocarbon age calibration curves, 0-50000 years cal BP. *Radiocarbon* **51,** 1111–1150 (2009).

11. Parnell, a. C., Haslett, J., Allen, J. R. M., Buck, C. E. & Huntley, B. A flexible approach to assessing synchroneity of past events using Bayesian reconstructions of sedimentation history. *Quat. Sci. Rev.* **27,** 1872–1885 (2008).
